# The post-war era in Nigeria and the resilience of Igbo communal system

**DOI:** 10.4102/jamba.v13i1.867

**Published:** 2021-04-19

**Authors:** Lawrence Okwuosa, Chinyere T. Nwaoga, Favour Uroko

**Affiliations:** 1 Department of Religion and Cultural Studies, The University of Nigeria, Nsukka, Nigeria

**Keywords:** Igbo, Biafra war, communal system, values, resilience

## Abstract

The Igbo people survived a civil war that raged between 1967 and 1970 and that devastated their land and reduced their population because of more than three million deaths. They were confronted with the challenges of beginning life afresh from scratch with almost nothing. Since then, they have allegedly been marginalised on a continuous basis by the Nigerian government. This notwithstanding the people with their communal spirit, which saw them through the civil war, have continued to cement their survival resolve in the post-war era. The aim of this article was to study the Igbo communal system as the bedrock of Igbo progress, especially in the past 50 years and recommends it as the basic principle of Igbo survival in Nigeria. It considers Igbo communal spirit as a veritable panacea against the recent agitations for secession by the people as that would guarantee Igbo people an ample space to operate in Nigeria. The methodology used in this article is a qualitative phenomenological method. This was carried out by interviewing some members of Igbo society, observing and interpreting events in Igbo society and as documented in literatures. It was found that Igbo people have really done well for themselves despite the seeming marginalisation by sticking to their resilient spirit. This study concluded that instead of seeking for independence from Nigeria, the Igbo people need to be mindful of their resilient communal spirit and reinforce it in all spheres of life. This would make them more relevant in the country’s affairs than they are currently.

## Introduction and background

On the 15th of January 1970, the Biafran War, also known as the Nigerian Civil War, which began on 6th of July 1967 between the government of Nigeria and the secessionist state of Biafra, came to an end, with the Igbo people being devastated. During that time, the Nigerian government made attempts to rehabilitate the people and the region in what has been termed, ‘the rhetoric of “No victor, No vanquished” and “Reconciliation, Reconstruction and Reintegration” (the 3Rs)’ (Ibeanu, Orji & Iwuamadi 2016:16) without much impact. Fifty years after the war, the people of Igboland still claim that they have not been fully integrated into the Nigerian society. They claim that they are systematically excluded from the affairs of the country, especially in government appointments, setting of infrastructures and in the sharing of the resources. The dissatisfaction amongst the people of Igboland over the state of governance in Nigeria has renewed the agitations for self-determination and secession. This buttresses what Walter (2004) said about wars that inflict high costs on people that they could exacerbate animosity and create a strong desire for retribution even after the war ends. She, further, said that grievances and divisions associated with such wars may be so intense that they are unlikely to subside long after the wars.

This seems to be the situation of Igbo people with regard to the Nigeria-Biafra War. Many Igbo people have not forgotten their sufferings and have proffered different solutions to their alleged marginalisation. For mainstream Igbo cultural organisations such as Ohaneze-Ndi-Igbo, Aka Ikenga, Mkpoko Igbo, Eastern Mandate Union (EMU), Odenigbo Forum, South East Movement (SEM), Igbo National Assembly (INA), Ndi Igbo Liberation Forum, Igbo Salvation Front (ISF), Igbo Redemption Council (IRC), Igbo People’s Congress (IPC) and the Igbo Question Movement (IQM), on the one hand, the solution is not in secession but instituting ‘true federalism’ as a national stabilising factor and enabling situation for Igbo socio-political growth.

On the other hand, there are some Igbo people who want nothing less than outright independence from Nigeria. At the forefront of this desire are two prominent associations. The first one is the Movement for the Sovereign State of Biafra (MASSOB), which was established after Nigeria’s return to democratic rule in 1999 by Chief Ralph Uwazuruike, who holds degrees in Political Science from Punjab University, India and Law from Bombay University, India. Later on, another group, with the same agenda, known as Indigenous People of Biafra (IPOB) emerged in 2012. The leader of this group is Nnamdi Kanu, a British Nigerian. It is an international organisation that unites many Igbo communities, particularly on the issue of seceding from the Nigerian state. Although the two groups had said their campaign was non-violent, their protestations and sit-at-home in Igboland at different times, have degenerated to violent clashes with the Nigerian armed forces and wanton destruction of lives, properties, economic activities and massive displacement of people (Ibeanu et al. 2016). Hence, on Friday, September 15, 2017, the Nigeria military declared IPOB a ‘militant terrorist organisation’ and, similarly, the South-East Governors forum, comprising of the five states of the region, proscribed the activities of the IPOB, to stop the rising tension in the zone (Chime-Nganya, Ezeji & Ezegwu 2017).

In view of the above-mentioned situation, this study looks at the alleged marginalisation of the Igbo people since the end of the war in 1970. It studies and brings to fore the communal resilience of the people to improve themselves and their land despite the alleged marginalisation. It examines the consequences of the recurrent agitations for Biafra and argues that Igbo people can still use their communal system to champion their cause against all odds instead of secession. Igbo communal spirit will strive in Nigeria, where rule of law is enthroned. Instead of secession, what Igbo people need to agitate for is greater political, economic and social inclusion in Nigeria. Igbo secession from Nigeria will limit their area of engagement and operation and could diminish their communal resilience as well.

This article adopts the qualitative phenomenological method by using direct observation, oral interviews, discussions and interactions with the interviewees and literatures.

### Theoretical framework

This work examines the concept of cultural resilience and its relation to communal survival and empowerment of people in an unfriendly polity. Cultural resilience is defined as ‘the use of traditional life-ways to overcome the negative influences of oppression, abuse, poverty, violence, and discrimination’ (Strand 2003:6). In other words, it considers how cultural background such as culture, cultural values, language, customs, norms, helps individuals and communities to overcome adversities and realise themselves. According to Clauss-Ehlers (2010):

The notion of cultural resilience suggests that individuals and communities can deal with and overcome adversity not just based on individual characteristics alone, but also from the support of larger sociocultural factors. (p. 6)

A culturally focused resilient adaptation is more than just about well-being and development of a people. For Panter-Brick (2015) it goes beyond these, instead it has important moral, social and political dimensions. It is about dignity, social justice, respect and self-actualisation.

Cultural resilience is nurtured from childhood, when children begin to identify their socio-cultural group members through the sharing of cultural values, experiencing discriminations from other groups, developing defensive mechanism against these discriminations and protecting their group’s interest.

### Post-war Igbo misfortunes in Nigeria

At the end of the Nigeria-Biafra War that raged between 1967 and 1970, Igbo people as defeated people incurred huge loss in all spheres of life and forced back into the union membership of one Nigeria. Although the war ended on ‘no victor, no vanquish’ verdict the clear indication that the people lost the war is obvious. An estimated three million Igbo people died. About 40% of those killed were Igbo children, either by gunshot or through starvation (Pellissier 2015). Some women carried the burden of shame for seeing their children die on their backs and their womanhood being assaulted by the rampaging victorious Nigerian army. The men folks were not left out in the shame of defeat as many who survived the war were frustrated for the rest of their lives. The people were debased and their sense of dignity was trampled upon so that somehow life seemed meaningless for those, who survived the war. There was no family that did not lose a person, had one misfortune or the other. Those who survived the menace and brunt of the war were roundly devastated. There were many wounded and diseased people who needed medical attention that was lacking. In the words of Njoku (in Obi-Ani 2009) the people were:

Thoroughly demoralized, psychologically disoriented, materially impoverished and politically marooned. Their future appeared permanently blighted. To be Igbo became taboo, and some Igbo groups attempted to hide Igbo identity by disguising their Igbo name. (p. vii)

Besides the human loss and its accompanying shame, the civil war destroyed landscape and infrastructures, thus leaving Igboland materially devastated. It put to a standstill any meaningful social and economic activity. Prior to the civil war, Igboland witnessed a great level of development. It was the home of the first university in Nigeria (University of Nigeria Nsukka). Its coal industry and its agricultural ventures were functioning. Healthcare delivery was also functional but with the war everything was grounded. Even feats achieved during the war like locally built petroleum refineries, Uli Airport, Ogbunigwe armoured car and other ingenuities were destroyed and never improved upon by the Nigerian government. Ezeani (2013), writing on this Igbo loss, stated that ‘In Biafra Africa Died’ because Igbo achievements during the war could have helped to develop the African continent.

The situation of the Igbo people worsened with the immediate expatriation of Christian missionaries in the area after the war by the Federal Government of Nigeria. This endangered the lives of many orphans and widows the missionaries catered for according to Mike (2019:oral interview). Unemployment level was so high and it was compounded by the fact that only 34 000 Igbo people were re-absorbed out of over one million unemployed people into the civil service (Obi-Ani 2009). Igbo people also believed that some of the laws enacted after the war were made to disenfranchise them in Nigeria, such as:

- The Public Officers (Special Provisions Decree no. 46 of 1970): With the Decree many Igbo officers who participated in the civil war on the part of Biafra were summarily dismissed or compulsorily retired (Federal Ministry of Information 1971). This was against the earlier directive and assurance to the world by the Head of State that all officers would be reabsorbed to their former positions before the escalation of hostilities.
- The Banking Obligation (Eastern States Decree): Banks in the Igbo region were made to pay all account owners a flat rate of 20 pounds independent of what they deposited in the banks before the war.
- The Indigenisation Decree of 1972: With this law, Nigerians were given an opportunity to get involved in the country’s productive enterprises. Igbo people, because of their post-war situation, feel they were not ready for such exercise and were alienated from the nation’s economy.
- Abandoned Property Policy: This policy of confiscating properties in the Rivers state by the state government was seen as an economic attack on Igbo people, who fled the state during the war.
- Igboland, which used to be one of the three major regions of the country, became the region with the least number of states of the six geopolitical zones in the federation.

Some Igbo people also see their defeat as not only political and economic but also religious. According to Chika (2019:oral interview), the people waged the war with almost nothing. They trusted in their deities to vindicate them against injustices they suffered. This is in consonance with their belief in *Ofo*, the Igbo traditional symbol and principle of justice and truth (Ezeanya 1967). Unfortunately, the war the people termed *Oguejiofo* [the war of justice and truth] was lost. This created a religious vacuum and crisis of faith amongst the people. Whilst according to Ugwu (2014), this situation put to question the demise of the Igbo gods; for Chika (2019:oral interview) it speeded the process of religious pluralism in Igboland.

## Methodology

The study adopts a qualitative phenomenological method in the sense that data were derived through a thorough desk review of existing literature that addresses questions relating to the study. In addition, interviews were conducted targeting 18 Igbo indigenes (refer to Table 1) who were purposefully sampled based on their knowledge of the subject matter, membership of some of the Igbo groups listed here and positions in their towns. The age range of the interviewees was from 25 to 80 years. This gave the opportunity of getting the views of those who saw the Biafra War and those who did not and are eager to have an independent Biafra country. The views and perspectives garnered from these interviews facilitated the refinement of the study’s assumptions and provided evidence to interrogate these assumptions. This study was carried out in the following Igbo towns: Oguta, Owerri, Mbaise, Nsukka, Umuahia and Awka. They cover four of the five Igbo states of the southeastern Nigeria. The thematic method of analysis was mostly used in analysing the qualitative data gathered in this study. A careful reading of the transcribed document was done to generate a logical and understandable analysis.

**TABLE 1:** The socio-demographic information of participants (pseudonyms).

S. No.	Name of participant	Sex	Status	Age (in years) when interviewed	Location when interviewed
1	Mike	M	Retired civil servant	67	Oguta
2	Chika	M	Retired civil servant	70	Oguta
3	Linda	M	Lawyer	45	Oguta
4	George	F	Farmer	60	Nsukka
5	Anya	M	Cleric	55	Nsukka
6	Nkechi	F	Lecturer	52	Nsukka
7	Christian	M	Cleric	65	Awka
8	Blessing	F	Unemployed university graduate	25	Awka
9	Pete	M	Youth leader	40	Awka
10	Esther	M	Politician	55	Umuahia
11	Ike	M	Youth leader	37	Umuahia
12	Chidera	M	Trader	66	Umuahia
13	Andrew	M	Politician	56	Mbaise
14	Meche	M	Civil servant	50	Mbaise
15	Onyi	F	Retired teacher	80	Mbaise
16	Oke	F	Women leader/politician	62	Owerri
17	Okoroafor	M	Business man	70	Owerri
18	Ogbe	M	Banker	48	Owerri

S. No., serial number; M, male; F, female.

### Data collection

**FIGURE 1:**
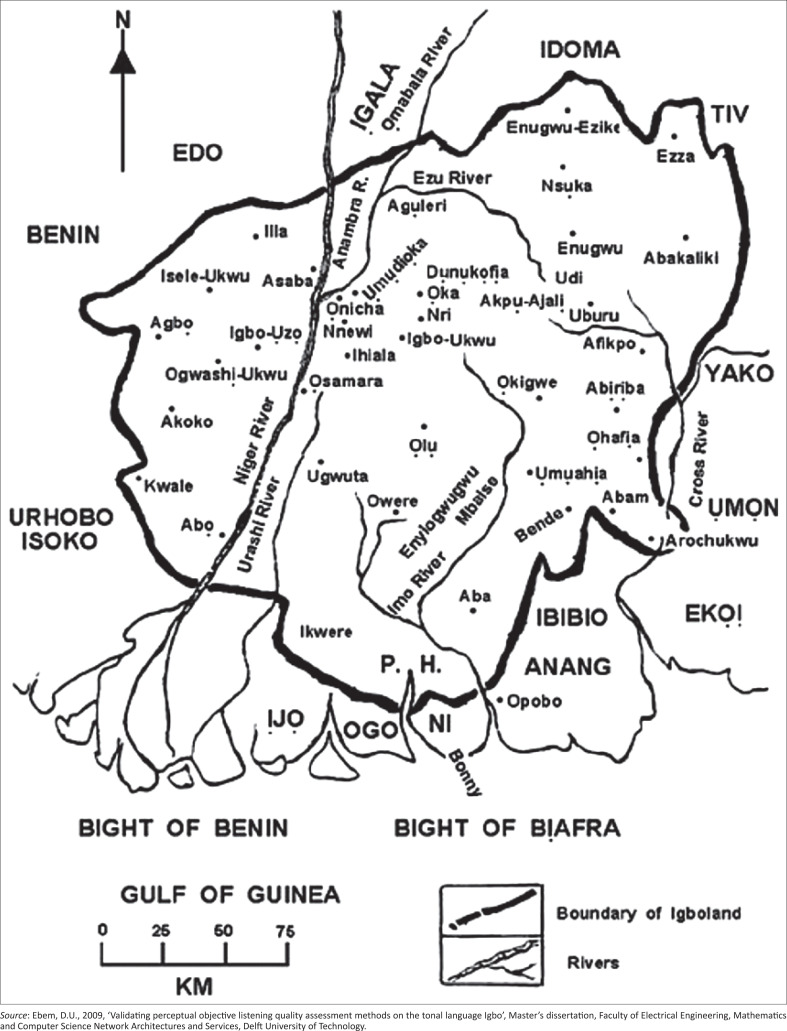
Map of Igboland in Nigeria.

The primary technique for data collection was the key informant interview. This was carried out through face-to-face interviews with informants in Oguta, Owerri, Mbaise, Nsukka, Umuahia and Awka. These areas comprise important parts in Igboland as seen in Figure 1 (Map of Igboland in Nigeria). The interviews were conducted in English with informants’ permission.

### Findings

As part of the methodology, individuals were interviewed and focus group discussions were held using unstructured interview questions. The salient points that were raised during these sessions are:

#### Igbo people are marginalised and victimised in Nigeria in different ways

All the people, who were interviewed privately or participated in focus group discussion, were in agreement that Igbo people are marginalised and victimised in Nigeria. They gave different reasons for this dismal situation. Some of the reasons are the perceived Igbo masterminding of the first military coup that saw many prominent northern politicians being killed (Linda, Ogbe & Andrew 2019:oral interview). Some participants argued that the Nigeria-Biafra War, which the Igbo people lost pitched them against all the other parts of the country (Esther et al. 2019:oral interview). Others maintained that Igbo people are naturally loud, adventurous and proud people, and this does not go well with other ethnic groups in Nigeria (Ogbe et al. 2019:oral interview).

#### All the participants maintained that Igbo marginalisation became obvious immediately after the war

According to them, Igbo people after the war were dispossessed and subjugated to all kinds of dehumanisation. What remained of the people was their life, which they buttressed in these Igbo names: Ndubuisi [life is supreme], Ndukuba/Ndukaku [life is more precious than wealth], Ndudi [with life there is hope], Ndubueze [life is king], Nduma [life knows all], Ndulaka [life decides all], Ndubuizu [life is all] and many others. Holding onto life, Igbo people started the journey of their survival with the full assurance that because God has kept them alive, He would sustain them because Chibundu [God is life], Chinwendu [God owns life], Chilendu [God is life’s provider] and Chikwendu [God wills life].

#### Igbo people have not really tackled the problem of their marginalisation well

Some people argued that the call for secession is not favourable to the Igbo cause (Mike et al. 2019:oral interview 2019). Some others believe that Igbo people have failed to align with the mainstream ethnic groups in Nigeria as they want to be alone (Andrew et al. 2019:oral interview). In general, they believe that Igbo people have not performed well to solve this problem.

#### Violent protests and agitation have helped to impoverish Igboland

The frequent demonstrations by pro-Biafra activists and their clashes with security agencies, according to most of the interviewees have adversely affected economic activities in Igboland. In some cases, the few infrastructures in Igboland have been destroyed or rendered non-functional. They further stated that the situation discourages investment in the geo-political zone as investors are wary of the situation.

#### Igbo attitude and lifestyle as contributing factors

Some of the interviewees mentioned that Igbo people’s lifestyle and attitudes, which includes pride, financial smartness and loudness have led to other ethnic groups’ mistrust of Igbo people (George et al. 2019:oral interview). They accuse them of over-domineering.

#### Internal division and backbiting amongst Igbo political class

Because of the perceived marginalisation of the Igbo people, some of the interviewees pointed out that Igbo people are almost undoing themselves with the intention of appearing as loyalists to other ethnic groups (Ike et al. 2019:oral interview). The quest to be accepted amongst the political class has led to internal division and lack of communal commitment amongst the Igbo political class.

#### Igbo people are naturally resilient

All the interviewees agreed that Igbo people are naturally resilient because of their communal system, which helps them to stand out against all odds and challenges. The general agreement on this is not without variations. The different perspectives to this affirmation are:

#### Igbo communal system is deeply rooted in their traditional cultures and values

Igbo communal spirit is inherent in Igbo people (Onyi et al. 2019:oral interview), for this, Nwosu (2014:379) opined that: ‘some commentators who are familiar with institutionalized monarchy describe the indigenous Igbo polity as “incomprehensible”’. It is not cyclical with a communist tincture. It does not require equity and equality in all facets of human life because personal aspirations beyond the confines of the communal cycle are acceptable and not seen as unworthy ventures. Every Igbo person has a divine genius or double in him or her – a personal god *Chi*, which helps the person achieve success in life. According to Okeke, Ibenwa and Okeke (2017):

The essential role of Chi concept is seen in the Igbo proverb, which says *Onye kwe, chi ya ekwe* (when one says yes, his personal god says yes) … Chi is a force in Igbo social behavior, which is characterized by an attitude of man; every being is the architect of his own fortune. In spite of the fact that the Igbo believe that the individual is the maker of his own fortune, they also believe in predestination, for they also agree that one’s Chi refers to one’s luck, which is associated with his destiny. (p. 2)

On the other hand, Igbo communal system is not lineal or pyramidic in form. The latter encourages survival of the ‘fittest or winner takes all’ mentality and exclusiveness at the top; whilst the former encourages steel rooted dogmatic hierarchy without any possibility of change in status quo. Igbo communal system, in contrary, can best be described as an inverted pyramid. Like the pointed base of an inverted pyramid, Igbo people’s base is small but strong. The people’s claim of brotherhood or being a community serves as a strong bond or launching pad into life. The pointed base does not give opportunity to any kind of domination amongst the people. No one is short-changed or deprived of his or her aspiration in life. It is about excelling according to one’s abilities and *Chi*. For this, Igbo people have expressions such as Igbo *enwe eze* [Igbo people have no king], *odibo feechaa eze eze ruo ya* [a good servant can become the king tomorrow], *nwata kwuo aka osoro okenye rie nri* [a hard working child dines with the elders], *ebe onye oso ruru onye ije ga eru ya* [with determination slow racers would still meet the fast ones at the end], *egbe bere ugo bere* [empathy – live and let live].

The story of the Igbo slave boy Equiano (2007) clearly illustrates the Igbo determination and will power to achieve success notwithstanding the circumstance. In his autobiography, Equiano gives credit to the widely held belief that Igbo resilience is nurtured from childhood. It is what every Igbo child imbibes by identifying and sharing Igbo cultural values. He writes about how he was kidnapped at the age of 11 from his village in Igboland. Although he was destined for an important role in his community, he did not allow his captivity to dampen this belief or alter his identity, self-awareness and vocation to greatness. He had to work very hard to purchase his freedom at the age of 21, something that almost every slave undoubtedly dreamed of; he was no longer the property of another. He captured the beautiful moment of his transition from being a slave boy to being a free person in these words (Equiano 2007):

When I went in, I made my obeisance to my master, and with my money in my hand, and many fears in my heart, I prayed him to be as good as his offer to me, when he was pleased to promise me my freedom as soon as I could purchase it. This speech seemed to confound him, he began to recoil; and my heart that instant sunk within me. ‘What’, said he, ‘give you your freedom? Why, where did you get the money? Have you forgot forty pounds sterling?’ ‘Yes, sir’, I answered … Accordingly, he [*his slave master*] signed the manumission that day; so that, before night, I who had been a slave in the morning, trembling at the will of another, was became [*sic*] my own master, and completely free. I thought this was the happiest day I had ever experienced. (pp. 100–102)

This acquired freedom notwithstanding; he had to face discrimination and persecution as a free black man. Life was never easy for him as a free black person but then he never gave up and had to make the best out of his situation. He went on to write his autobiography and committed himself to the abolitionist cause and published his Narrative.

Obviously, Igbo communal system is antimonarchy. The people express this antimonarchy by saying that *ezebuilo* [a king is an enemy] and that *Igbo enwe eze* [Igbo people do not have kings]. In fact, Igbo people are radical republicans or ultra-democratic in their social and political organisation. Sometimes, this is interpreted erroneously to mean that Igbo people are intensely individualistic. On the contrary, the idea of ‘community’ or ‘communal value’ is not only lacking but is also meaningful to the Igbo people. In Igbo cultural setting, the community is supreme. In fact, every Igbo person is an embodiment of his family and community. In the words of Ezekwonna (2005:204) ‘he (the Igbo person) owes his existence to the past generation, the ancestors and his contemporaries. He remains without doubt part of the whole’. The community is a source of strength, greatness and success for the Igbo person. Hence, an individual’s success is seen as that of the community. The success of an individual does not extricate the individual from the community because the community’s approval is always necessary and sought for. Although the individual strives for personal glory, according to Okeke (2006:32) such an individual must receive social recognition or else it is worthless. On the face level, Igbo people may seem individualistic and independent of the community, no Igbo person can feel fulfilled without the community.

Igbo inverted pyramid communal system is like a wake-up call for all the people. In this structure, no person, no village or town is left in the ‘waking up’. It encourages hard work and healthy competition amongst individuals and amongst communities. In the same vein, ‘Igbo’s concern for hard work and achievements tend to leave them with little patience for the lazy and for failure’ (Oguejiofor in Ezekwonna 2005:102). For this, Igbo towns, families, groups and individuals do everything in their power to uplift themselves, especially those who are specially talented and dotted with special gifts. The logic is that their success would 1 day benefit the entire community. A clear example is the story of Obi Okonkwo in *No Longer At Ease*. In this novel, Achebe (1987) narrated how the members of Umuofia Progressive Union (UPU), Obi’s kinsmen living in major Nigerian cities, together raised fund to send him to England to study law, in the hope that he will return to help his people.

Igbo communities are known to engage in developmental projects in their different communities. According to Uchendu (1965):

[*T*]here is no Igbo village today which cannot point with pride to a motor road, water points, a marketplace, a village school, a maternity centre or a village hall or even a combination of these as the result of their own efforts. (p. 36)

Some Igbo communities have built schools, hospitals and post offices and handed over the same to relevant authorities for management. To alleviate the suffering of individuals and create bond amongst the people, Igbo communities such as Oguta and Akpodim in Imo state, Awgu in Enugu State, Mbutu Ngwa in Abia state and Ihiala in Anambra state have communal lands for farming and other activities.

- **Igbo concept of *Onyeaghala nwanne ya*** helped the Igbo people to grow together even in their adversity (Nkechi et al. 2019:oral interview). The aforesaid concept shows that extended family and community are of utmost importance in Igboland. In Igboland, ‘to be’ is to be part of an extended family (*nwanne* – literarily meaning mother’s child i.e. sister or brother). Interestingly, mother here does not mean the biological mother but the earth goddess – *Ala. Ala* is the mother of all Igbo people, from her comes forth and returns the Igbo people. Without Ala, no Igbo person would ever achieve anything meaningful in the land because on her is the human person born into, walk, plant crops and erect a shelter. She is sacred and the eternal home of the Igbo people. It is where the ancestors return to and, for this, libation is poured on the land for them to partake in the people’s life. Hence, *nwanne* as extended family member does not mean that the Igbo people resemble one another, speak the same language, behave in the same way but simply are offspring of the same *Ala*. They share common fate and experience as a people. For this, Igbo people wherever they see themselves, especially in foreign lands, where they are likely to be vulnerable and faraway from Ala’s protection see themselves as *nwanne* [brethren].
- **Ahamefuna** is another concept that portrays Igbo communal life (Ike et al. 2019:oral interview). For Iheanacho (2016)
  Ahamefuna theory is anchored on the Igbo belief that their names should not be erased from the kindred or village because of inability to produce a male issue to continue one’s own lineage. (p. 105)

Parents give their wards this name as a prayerful wish over them to procreate and promote their lineage. This is a great source of motivation for Igbo people in their daily endeavours. As a concept, *Ahamefuna* reminds Igbo people of their origin, identity and communal expectations:

- The principle of *Igwebuike* is important in the resilient communal spirit of the Igbo people (Anya, Nkechi & Okoro 2019:oral interview). Literarily it means ‘Group/Community is strength’ but could easily be translated as communal strength or collaboration and unity is supreme. The principle has so much in common with other Igbo concepts such as *Nwanne* and *Umunna*. According to Iroegbu (Kanu 2015) this communal strength is characterised by a common origin, common world view, common language, shared culture, shared race, colour and habits, common historical experience and a common destiny. Whilst for Kanu (2015) *Igwebuike* is the central factor in Igbo hospitality, friendship, honesty, solidarity, working together, respect for elders and life. Igbo communal success is hinged on *Igwebuike* because with it the community can surmount any difficulty. Here, community for the Igbo people includes local gods, ancestors, the living and even the unborn members (Ezekwonna 2005) and all have roles to play in the development of the community. The gods provide good health, enabling environment and blessings for any project to succeed in Igboland. The ancestors are responsible for protecting their wards from the menace of evil spirits, unseen enemies and the maintenance of intra family peace. It is only when these are in place that human efforts become meaningful. For this, Igbo people say that *Chi onye na edu ya* [One’s personal god guides one].
- **The belief in *Ikenga* as motivational** (George & Mike 2019:oral interview) Igbo people’s belief in *Ikenga*, the symbol of Igbo philosophy of industry and strength, success in trade, war, hunting and farming (Ezeani 2013; Talbot 1926) is considered one of the factors that influences Igbo resilience. According to Ihediwa (2014):

This force is reputed to have mediated in the affairs of men and assists its owners to achieve success in their chosen endeavours … The possession of *Ikenga* depicts high status, accomplishment, wealth and integrity in the society. However, it is mostly owned by warriors and great men; but individuals aspiring for upward mobility in life and the poor also could own it and look upon it for breakthrough in life. (p. 6)

Spiritual offerings were made to activate the potency of the *Ikenga* in people’s life. True or false, the psychological motivation and spiritual blessings derived from this belief worked for the people.

The belief in *Ikenga* includes not accepting defeat. Notwithstanding the outcome of the Biafran War, Igbo people do not see themselves as war losers. They feel themselves proud fighters because the war ‘brought out the best in the Igbos (sic) endurance, creativity, adaptability, indomitable will to be, sense of solidarity, sense of common cause, collaboration and vigilance’ (Uwalaka 2003:16). Whatever happened was simply their destiny, which Opata (1998:165–166) captured very well in these words: ‘The immediate post war years in Igbo land found many young boys and girls suddenly resorting to this epithet: o so m?’ Literally, this translates to: ‘does it follow me?’ In actual sense, what the statement means is: ‘is it my fault’ or ‘am I responsible for it?’ With this kind of mental attitude, it was quite easy for the people to begin afresh knowing very well that the defeat of the war was not their fault. In fact, so many things worked against the emerging Biafra country. Local and international conspiracies were the worst culprits. In the words of Ezeani (2013:55): ‘Britain and its allies opted to support the Nigerian side of the conflict instead of the Biafra side, not minding which side was the aggressor and which the aggrieved’.

The belief in *Ikenga* psychologically boosted Igbo people to take risks and resist oppression. It reinforces the Igbo attitude to work that *aka aja-aja na ebute onu manu manu* [only a soiled hand can guarantee food]. In this way, Igbo people are always seeking for novelty and autonomy as requisites for survival in Nigeria. According to Okeke (2006) it makes Igbo people to feel equal with some people and superior to others but never inferior to any person. For Iwara, Amaechi and Netshandama (2019):

Their competitiveness and enterprising skills made them migrate in droves away from their cultural enclave in southeastern Nigeria, to other parts of Nigeria and beyond, where they have created and established different lines of enterprises. (p. 229)

Olutayo (1999), may not be aware that he was pointing to the Igbo people’s belief in Ikenga as a source of their resilience, when he affirmed that:

One major and unique trait of the Igbo entrepreneur is the courage, perseverance, and determination with which they carry on in spite of the bad experiences and losses during the Nigerian civil war from 1967 to 1970. This … is at the heart of the apprentice system which ‘brings an ethic of denial, hardship and discipline’ gathered through trading experience which is itself risk-prone. With little or no government assistance, the Igbo have moved from trade to industry since the end of the civil war. Most of these new industrialists possessed elementary education, apprenticeship, and trading experiences before they undertook their industrial venture. (p. 164)

The resilient spirit of the Igbo people, according to Abaribe (2017) guaranteed that within a short time after the war, almost all the important markets in Lagos state, as in other states of the federation, were in the hands of the Igbo people. The combined turnover daily of these markets run into billions of naira daily.

### Igbo women’s participation

The traditional role of women in Igboland changed during and after the war showcasing their fair share of the resilient Igbo communal spirit (Okoro, Nkechi & Anya 2019:oral interview). Before the war, Igbo women were mostly confined to domestic affairs of training their children and looking after the compound, doing petty trading and farming, pottery making, spinning, weaving, basket work and grass plaiting. But with the war, this changed. During the war, some Igbo women joined the Biafran Army as cooks, nurses, spies and some traded in contraband goods across the warfronts to make ends meet. One of those interviewed, Blessing (2019:oral interview) interpreted it as the reawakening of the spirit of the Aba Women’s Riot of 1929 against British oppression of Igbo women. This spirit made Igbo women to stand up against the social, political and economic oppression faced after the Biafra War. Igbo women became more adventurous like their men counterpart and engaged in all kinds of businesses to help their families and community. Today, they are known to organise themselves as *Umu Ada* [Daughters] and *Ndi Inyom* [Wives] for developmental purposes in Igboland. Their annual August Meetings are geared towards the same purpose.

### Communal competition

One of the factors that aids Igbo resilient communal spirit is inter-communal competition (Linda et al. 2019 :oral interview). As the individual derives his or her identity from the community, in Igboland, communal success overrides individual success. In view of this, individuals work hard to bring to limelight their communities and make them the envy of others. In fact, as soon as an Igbo person starts making money, he or she is bound to look homewards. The person wants the community to recognise him or her and for the community to do so, the person must have contributed meaningfully to the development of the community. This social responsibility has helped immensely in rebuilding the devastated Igboland. This is in line with what Nwafor-Ejelinma (2012) said:

The indomitable resilience of the Igbo identity became very manifest after the civil war. Towns and institutions that were maliciously destroyed or completely obliterated by vandals in Nigerian military uniforms during the civil war were quickly replaced with structures of ultra modern architectural finesse. Places like Onitsha, Ukpor, Ogidi, Awka, Enugu, Abakaliki, Owerri, Aba, Abagana, Umuahia, Nsukka, Port Harcourt, Okigwe, Aguleri, Asaba, Uguta [*sic*] just to mention a few that were badly hit, now look much better and as if nothing did ever happen. (p. 43)

This transformation is very remarkable that a British journalist, who visited the war-torn zones, Onitsha, Enugu, Aba, Owerri, Umuahia, five years after the war, precisely in 1975, said: ‘Nothing can surpass the resilient spirit of the Biafran people’ (Nwafor-Ejelinma 2012:43):

- **Profit without morality:** Some of those interviewed, about 30%, affirmed that Igbo resilience as practiced today is not always in line with core Igbo values (Anya et al. 2019:oral interview). They allege that because of the claim of marginalisation, some Igbo people do anything possible to survive. In a way, it has become for them a race for the fittest without morality. Unfortunately, this is in contrast to what Panter-Brick (2015) pointed out of the resilient cultural spirit. Such a spirit needs to profit not only well-being and development but also social justice, respect and self-actualisation.
- **Suggestions:** Amongst the major suggestions on the way forward for the Igbo people by those interviewed are the need for Igbo people to be conscious of their resilient nature and thus, put a resilient structure that would help them participate as equal partners in the Nigeria polity (Andrew, Ogbe & Okafor 2019:oral interview). It was also suggested that if Igbo people are to seek independence from the country then it must be done in a peaceful way like calling for referendum (Linda et al. 2019:oral interview). Some interviewees suggested that Igbo people return to their core cultural values and invest more in Igboland (Mike et al.). Only two interviewees suggested secession as the way forward for Igbo people (Ike & Pete 2019:oral interview).

### The resilience of Igbo communal spirit after the war

Notwithstanding the defeat suffered at war and the prevailing unjust situation, Igbo people have not failed to contribute concretely and meaningfully to the Nigerian project. In spite of the challenges they face, they have made enormous progress. They have risen from their subdued position to become one of the most educated Nigerians; the largest group of domestic investors in the country, the next largest group after the indigenous population in all parts of Nigeria and the richest and largest pool of Nigerian diaspora population (Abaribe 2017). These achievements can be attributed to Igbo people’s communal spirit. This survival spirit energises the quest to be like others if not better than themselves. The communal spirit, which encourages everyone to give his or her best helped in Igbo people’s path of recovery.

According to Linda (2019:oral interview): ‘Igbo resilience is not usually violent or destructive except when they are pushed beyond their limits’. It is built on a great sense of self-worth and pride, which the people express in the adage:

[*O*]*nye ajuru anaghi aju onwe ya* (a rejected person does not reject himself or herself), they hardly accept defeat even if it takes many years to prove it. They will always look for alternatives to prove their worth.

This understanding could explain what happened in Igbo Landing, a historic site at Dunbar Creek on St. Simons Island, Glynn County, Georgia. Boakye (2018) narrated the story in this way:

Historians say Igbo captives from modern-day Nigeria, purchased for an average of $100 each by slave merchants John Couper and Thomas Spalding, arrived in Savannah, Georgia, on the slave ship the Wanderer in 1803 … The Igbo were known by planters and slave owners of the American South to be fiercely independent and more resistant to chattel slavery. According to Professor Terri L. Snyder, ‘the enslaved cargo “suffered much by mismanagement,” “rose” from their confinement in the small vessel, and revolted against the crew, forcing them into the water where they drowned’. Led by their chief, the Africans then marched ashore, singing. At their chief’s direction, they walked into the marshy waters of Dunbar Creek, committing mass suicide. (n.p.)

Linda (2019:oral interview) further said that many Igbo people because of their desire to climb the social ladder and not bemoan their fate in self-pity had to migrate to other African countries such as Ivory Coast, Gabon, Cameroun and Togo and later to the western world in groups in search of greener pastures. Although, it has never been easy for them both at home and diaspora, Igbo people have continued to distinguish themselves in different fields of human endeavours, besides the few of them, who indulge in fraudulent ways to survive.

The desire to be respected and accorded special position in the *scala pyramida* played a great role in repositioning Igbo people after the Biafra War. No family or person wanted to be left behind. Most people knew that remaining focused and working hard were the only options left for them after the decimation of the war. In view of this, they did all they could to succeed.

In Igbo culture, title-taking and exhorted positions are acquired and not given freely. According to Onyeozili and Ebbe (2012:31), ‘The Igbo respected and honor achieved status more than ascribed status. Individual achievements determine a person’s social position in his community’. These titles and positions are determinants of how industrious and successful a person is in life. They show that one is disciplined, mature, responsible and capable of helping others. As it is a legitimate aspiration for an Igbo person to climb the social ladder, the culture of hard work and discipline is in a way inculcated. According to Ezekwonna (2005), the underlying factor to this is not greed or avarice but eschatological. This is because (Ikenga-Metuh 1985):

They [*Igbo people*] have the belief that this world is a carbon copy of the invisible world. That is to say that all the acquired fame and status will be taken along to the next life … it is part of condition for one to be accepted as an ancestor in the hereafter. (p. 100)

Furthermore, because Igbo people are conscious of their place in the inverted pyramid, kind of communal life and its eschatological implication (Onyeozili & Ebbe 2012):

[*T*]hey are very assertive and proud of their achievements, and they raise their children not to fail in life. In effect, the Igbo material culture is engulfed in ingenuity and creativity. (p. 31)

Igbo people’s communal resilient spirit flourished with the systemic silence on the war in national dialogue and schools. According to Ejiogu (2013):

Biafra, and the tragedy it represents for humankind, is compounded by the official code of silence that Nigeria’s military decreed in the 1970s to ensure that the Biafra–Nigeria war is not taught in schools in Nigeria. (p. 741)

The Nigerian government lost the opportunity to inculcate nationalism and reintegration of the Igbo people by banning discussions and teaching of the war history in schools. According to Maiangwa (2016):

There are ‘no cemeteries, no graves marked, nor monuments – not even lists of the fallen’. The lack of public remembrance of the victims of the Nigeria-Biafra war may partly be attributable to the fact that there was no actual transition and reconciliation in the aftermath of the war. The government’s ‘no victors, no vanquished’ policy of reconciliation only served to heighten suspicion of its lack of readiness to address the memory and trauma of the war. As a result of the suppressed memory of the war, many Igbos [*sic*] and easterners have been left with a deep sense of injustice. (pp. 53–54)

This systemic silence made Igbo people to deal with the war’s raw feelings in their own way. They narrated the story in their own way to their wards. In fact, what Igbo children heard after the war was more of the bravery, sacrifice and ingenuity of their parents. An example of such narration is (Harnischfeger 2011):

‘We survived three years of blockade […]. We were able to build our own rockets hitting their targets with precision. We had our means of getting things done. We refined petrol and brake fluids from coconut’. ‘Since then, no other Black race has done it. […] During the time, our own airport was the busiest night airport on the continent of Africa. We did it. […] The range of our broadcast was fabulous. Practically, the whole continent of Africa was hearing the Voice of Biafra. And it came from the back of a lorry. […] the great thing about Biafra was that everybody was working for everybody else. That was a great thing. There was no stealing’. (p. 101)

Other stories include the all-conquering ogbunigwe and the capsized Nigerian army gun boats in Oguta Lake. These stories make Igbo people, especially those born after the war, to see the war as a mark of patriotism to Igboland and as a lost opportunity for freedom and development. For them, it is a worthy venture to cue into their parents’ sacrifice for Igboland. This explains their attraction to MASSOB and IPOB. This situation has helped to promote Igbo nationalism and not patriotism to Nigeria.

The alleged Igbo people’s placement of economic activities above other human endeavours is not without a cause. Their unimaginable suffering, during and after the war, helped the people to rediscover themselves, their limitation and position in the country. They realised that they could not compete favourably with other ethnic groups in the country in terms of politics, infrastructures and agriculture because of their depleted population, devastated land and perceived injustice to them. To make ends meet, they imbibed the philosophy that ‘the economic activities of a people stimulate development, which, in turn, affects every aspect of a community, such as the history, politics, legal system, religions and others’ (Nwosu 2014:157). Based on this, Igbo people are alleged to view reality from an economic standpoint.

### Ethical considerations

This article followed all ethical standards for research without direct contact with human or animal subjects.

## Discussion

Even though the war has formally ended, Igbo people, according to Uwalaka (2003), still feel vanquished. All over the country, there are signs of a country battling with the effects of the war. In the words of Adujie (2011):

The events which precipitated and led to the Nigerian Civil War, the war itself and its aftermath have shaped and continue to shape the economic and political development of Nigeria. These events and the lingering ill-will, have poisoned relationships between various regions, religions and ethnic groups in Nigeria. These events have extraordinary adverse effects which have and continue to stunt and stymy Nigeria. (n.p.)

But the unnerving cries are coming more from Igboland. The people are under great pains. There appears to have been more insidious, more perfidious, more destructive and dangerous ‘war’ against them – the war of marginalisation and exclusion in the economic, bureaucratic, structural and political space of the country. This makes it difficult for the people to actualise their dreams and potentials to the fullest. They are made to feel impotent and disinherited in Nigerian affairs.

Since after the Biafra War, Igbo people have suffered more structural injustice than any other part of the country. Igbo people have been systematically edged out from the military hierarchy, civil service and core infrastructures. In the words of Ekwueme, former vice president of Nigeria (Uwalaka 2003):

The inequalities and injustices of states exercised by successive Nigerian governments have resulted in gross Igbo under representation in all federal institutions because of federal character principle based on equality of states … Igbo emasculation has come from population manipulation, boundaries adjustment and gerrymandering in the delineation of federal constituencies in the politics of 1970–1979 and 1983 to 1999. (p. 21)

In the present dispensation, Onwubiko (2017) captured Igbo marginalisation in this form:

Buhari had made over 100 top level appointments and the North have taken over 80% of these slots including nearly 90% of all the top 30 appointments into the Nigerian National Petroleum Corporation (NNPC). The President has left out the Igbo speaking population of Nigeria in all of these major appointments. (p. 1)

The attitude of the government does not only disenfranchise Igbo people from the affairs of the country, it sends bad signal to them that the war has not really ended.

In conjunction with the given discussion, Baiyewu (2017) noted that Ekweremadu, a former deputy senate president maintains that:

[*T*]he worst disadvantages suffered by Igbo people are not just those imposed by structural imbalances, such as fewer number of states and local governments or the lesser revenue accruals, political representation, federal employments and political appointments, arising from the imbalances and wilful [*sic*] injustice. The greatest marginalisation and disadvantage suffered by Igbo people is the wilful [*sic*] dissembling and discarding of true federalism, which the founding fathers of Nigeria adopted, in order to live together as one nation in which no one is oppressed and every component part is able to thrive. This awkward form of federalism (referring to present configuration) has boxed Igbo people to a tight corner and caged their potentials and ingenuity. (p. 1)

The statements from Ekwueme and Ekweremadu cannot be dismissed easily as both have held high positions in the Nigerian government. The Igbo have been weakened to the point that no matter how hard they try they are bound to fail. No matter how hard they try to bridge the gap between them and others, the gap is not diminishing instead a great gulf appears steadily and keeps widening. The Nigerian structure does not allow Igbo people to live out fully their communal life and attain the highest benefit from it. Again, it is not their fault (*o so m*?).

More than anything else, the worst hit in this ugly situation is Igbo ideals and principles, which are bastardised. The actual state of things in Nigeria has made some Igbo people to jettison their identity and cultural values and engage in nefarious activities in order to survive. Some Igbo youths are living with the euphoria of having their own country and thus have stopped living as Nigerian citizens. The situation has become so bad that Nwankwo (2000) stated that ‘the fate of Ndigbo (sic, same as Igbo people) has gone beyond marginalization and entered the dangerous state of alienation and exclusion’. Based on this, Igbo people need to re-strategise using their resilient communal spirit.

### The way forward

From the post-war experience of Igbo people, it is clear that the Reconciliation, Reconstruction and Reintegration promised them are utopic. This, unfortunately, has aggravated the Igbo quest for an independent nation. No matter how legitimate this ambition may be, it is good to point out that Igbo people have invested so much in the Nigerian polity for them to extricate themselves from it. From the suggestions of those who were interviewed, it is obvious that what Igbo people need most is a change of attitude and not that of a country. They are to engage themselves in the polity as true Igbo people, who are conscious of their identity and cultural values of respect for life and human dignity, promotion of respectable togetherness, hospitality and sense of being sacred. They are to be truly themselves by so doing they give their best. Igbo communal interest is to be at the basis of all their engagements. They can only be their own messiah by working together as depicted in Igbo ideology, *onye aghala nwanne ya* [no one is to be left behind].

Igbo identity and ideology are to be revitalised as stimuli for Igbo consciousness because ‘the mainstream of Igbo identity and personality was destroyed by the Biafran war’ (Harnischfeger 2011:100). The ways to this is for Igbo people to fathom what it means to be an Igbo person in the Nigerian context today. To imbibe the spirit of *Igwebuike* [community is strength], Igbo communal spirit that has always worked for the people in all spheres of their life. Whenever Igbo people come together, they have always remained the envy of others. The reverse is the case as evident today. Although Igbo people are highly successful individuals, they cannot create much impact on the Igbo people because they are not working together.

Igbo communal identity does not negate personal identity or interest but aligns it to the acceptable communal principles, which guides Igbo communal identity and interests. Amongst these principles are *ezi afa ka ego* [good name and dignity are worth more than wealth], *kama m ga-eri dachie uzo ka m gbara onu* [contentment], *ezi okwu bu ndu* [Truth is life – integrity] and *ome mgbe oji ka onye ohi mma* [discipline and honour]. With this, Igbo people would come to realise that ‘what unites the Igbo nation is not just a unique experience of suffering, but also a unique mission’ (Harnischfeger 2011:105). Igbo communal spirit is a mission that Igbo youths need to imbibe and exhibit in Nigeria. They are to refrain from violence and destructive tendencies and make Igboland a heaven of peace for investors.

Although Igbo marginalisation and exclusion from Nigerian affairs is despicable and detestable, secession may not be the way forward. This is because Igbo people, with their resilience spirit, are everywhere in Nigeria making them truly Nigerians. More than secession, Biafra is to serve as an ideology for Igbo people everywhere in Nigeria against any form of marginalisation, oppression and suppression. Biafra would stand for the sanctity of life and equal opportunity for all. Biafran ideology is to serve Igbo interest and collective reminder of Igbo principles of *Ozoemena* [let it not happen again] and *Echezona* [Let’s no one forget]. In this way, Igbo people would make themselves relevant in a country that is lacerated with injustice and devoid of rule of law. They would help in building a new Nigeria that would accommodate all and sundry independent of language, religion and ethnic group. In fact, what Igbo people and the entire Nigerian people need is a restructured country.

Igbo resilient communal spirit would truly make sense if Igbo people start looking homeward and invest in Igboland. As Igboland will always remain the homeland of Igbo people, it deserves all the attention it can get from Igbo people. This is in line with Igbo affirmation that *aku ruo ulo okwuo onye kpara ya* [when wealth is brought home, it speaks for itself]. This would help to change the tide, which Olutayo (1999) stated in this way:

Since the avenue available for mobility within the Igbo political economy is external to its social structure, it more or less became inevitable for the Igbo to migrate outside their communities. From there they engage in all sorts of activities, especially trade, in order to achieve the aim of survival and status mobility. (p. 165)

Igbo people can survive in Igboland and achieve their Biafran ideology in Igboland, if they invest more in Igboland.

## Conclusion

In this article, it has been highlighted that Igbo people are marginalised in the Nigerian polity and that the 3Rs, Reconciliation, Reconstruction and Reintegration, promised to the people of Igboland immediately after the war by the government have not been realised. Igbo people are facing a lot of challenges in the Nigerian polity, which has led to some of the people opting for secession even though they failed to achieve with the Nigeria-Biafra War. The main contention presented in this article is that even though Igbo people are not having it easy in the present Nigerian structure, Igbo people have performed well for themselves through their communal resilient spirit. Igbo people have made remarkable achievements in post-war Nigeria through their communal resilience. These achievements have already set them apart. Hence, instead of seeking for an independent country from Nigeria, the article contends that Igbo people should be mindful of their resilient communal spirit and put it into practice in all spheres of their life. This would make them more relevant in the country’s affairs than they are currently and if they communally work together, they would achieve more than they have achieved in the past. The quest for an independent country may limit Igbo people’s area of engagement, especially as the world turns to a global village.
